# Molecular phylogeny of *Culex* subgenus *Melanoconion* (Diptera: Culicidae) based on nuclear and mitochondrial protein-coding genes

**DOI:** 10.1098/rsos.171900

**Published:** 2018-05-23

**Authors:** Carolina Torres-Gutierrez, Tatiane M. P. de Oliveira, Kevin J. Emerson, Eduardo Sterlino Bergo, Maria Anice Mureb Sallum

**Affiliations:** 1 Departamento de Epidemiologia, Faculdade de Saúde Pública, Universidade de São Paulo, Avenida Doutor Arnaldo 715, CEP 01246-904, São Paulo, Brazil; 2 Research Associate, Programa de Estudio y Control de Enfermedades Tropicales, PECET, Facultad de Medicina, Universidad de Antioquia. Calle 67 No. 53-108, Medellin, Colombia; 3 Biology Department, St. Mary's College of Maryland, St. Mary's City, MD, USA; 4 Superintendência de Controle de Endemias, Secretaria de Estado da Saúde de São Paulo, Araraquara, São Paulo, Brazil

**Keywords:** Spissipes Section, Melanoconion Section, systematics, molecular phylogeny, neotropical species

## Abstract

The subgenus *Melanoconion* of the mosquito genus *Culex* is taxonomically diverse and is widely distributed in the Neotropical Region, with 10 species occurring in the Nearctic Region. Species of this subgenus pose a taxonomical challenge because morphological identification is based largely on anatomical characters of the male genitalia. We addressed the monophyly of the Spissipes and Melanoconion Sections of the subgenus *Melanoconion* and some of the informal groups in each section. Our sample taxa included 97 specimens representing 43 species, from which we analysed fragments of two single-copy nuclear genes (*CAD*, *HB*) and one mitochondrial gene (*COI*). Phylogenetic relationships within the subgenus are presented based on results of maximum-likelihood and Bayesian analyses using a multi-locus matrix of DNA sequences. We show a molecular phylogeny of *Melanoconion* in which both sections were recovered as monophyletic groups. The monophyly of the Atratus and Pilosus groups was confirmed. Within each section, other monophyletic groups were recovered highlighting the potential need for future nomenclature rearrangement. The phylogenetic signal contained in nuclear genes, when analysed together, was more informative than each gene analysed separately, corroborating monophyly of *Melanoconion* relative to *Culex* (*Culex*) species included in the analyses, the Melanoconion and Spissipes Sections and some species groups. Our results provide new information for the classification of the subgenus and additional data that can be used to improve species identification when a more representative taxon sampling is available.

## 1. Introduction

The subgenus *Melanoconion* is one of 26 subgenera of the mosquito genus *Culex*. The majority of its species occur in the Neotropical Region [1,2], in different ecological regions, which vary from conserved and pristine forests to highly modified, anthropic environments [2–5]. As blood feeders, *Melanoconion* species display a wide variety of hosts (birds, mammals (including rodents), reptiles), with many species competent to transmit viral pathogens to a wide spectrum of vertebrates that include humans. Several studies have documented species of the subgenus as potential and effective vectors of viruses of the Venezuelan equine encephalitis complex (including Venezuelan equine encephalitis virus, Everglades virus, Mucambo virus), West Nile virus and eastern equine encephalitis virus [5–13].

The subgenus includes 160 species [14], which are separated into two major sections: Melanoconion and Spissipes [15]. Each section is further divided into several groups and subgroups [16]. Sallum & Forattini [2] reviewed the Spissipes Section, and provided morphological identification keys to species, along with information on bionomics, medical importance and geographical distribution of all species included in the section. Unlike the Spissipes Section, there are no recent taxonomic revisions for species included in the Melanoconion Section, and the available literature provides little information on the relationships of the subgenus [16]. Several studies have supported the monophyly of *Melanoconion* [17–20]; however, their conclusions are based on a small number of species (ranging from 2 to 9) distributed within the subgenus. Another contribution to the study of phylogenetic relationships among species of the Spissipes Section tested only the monophyly of species included in the Vomerifer Group and Pedroi Subgroup [21]. Harbach *et al*. [22] did not find support for the monophyly of the subgenus *Melanoconion* when conducting a morphological analysis of 86 species of *Culex*, including three species of *Melanoconion*, that recovered a monophyletic lineage containing the subgenera *Melanoconion*, *Aedinus*, *Anoedioporpa*, *Tinolestes*, *Belkinomyia*, *Nicaromyia* and the genera *Galindomyia* and *Deinocerites*.

The search for appropriate molecular markers to infer natural relationships is an important step in any study [23]. Previous analyses have recommended single-copy nuclear genes [24–26]. Among eight nuclear genes explored for different invertebrate taxa, *CAD* (also called rudimentary) has been referred to as one of the most informative and easy to amplify genes [26–28]. Studies using nuclear genes of mosquito species have been used mainly for *Anopheles* species [29–32]. Studies by Besansky & Fahey [33] and more recently by Reidenbach *et al*. [34] have used protein-coding nuclear genes, including HuncHBack (*HB*) and *CAD*, to solve questions about the relationships of different groups of Culicidae.

Furthermore, the mitochondrial gene, *COI*, also referred as the DNA barcode for species identification, has been extensively used for taxonomic purposes in many vertebrate and invertebrate orders [35,36]. *COI* sequences have been also used to infer relationships in species of Culicidae [20,37,38]. Recently, results of studies using the barcode region of the *COI* gene showed that it is both a useful and accessible tool in the identification of species of the subgenera *Culex* [39] and *Melanoconion* [40]. In this study, we addressed the current classification scheme of the subgenus that is based on morphological features [2,15]. We addressed the monophyly of the two major sections of the subgenus and addressed relationships within each section using sequence data from two nuclear (*HB* and *CAD*) and one mitochondrial (*COI*) protein-coding gene.

## 2. Material and methods

### 2.1. Taxon sampling

In the study, representatives of the two sections of *Melanoconion* were included in the ingroup that consisted of 97 specimens of nine species of the Spissipes Section and 34 species of the Melanoconion Section (table 1; electronic supplementary material, table S1). The outgroup was composed of one specimen of *Culex* (*Culex*) *quinquefasciatus* and two specimens of *Culex* (*Culex*) *mollis*. The selection of *Culex (Culex)* species as outgroup taxa was based on the availability of specimens.

**Table 1.** List of formal species and possibly newly discovered taxa of the subgenus *Melanoconion* included in the study (with a three-gene matrix), taxonomic classification and number of specimens used in the analyses. The classification adopted herein is based on Sirivanakarn [15], with modifications by Sallum & Forattini [2].

section	group	subgroup	species	specimen (*n*)
Melanoconion				
	Atratus	—	*Cx. commevynensis*	2
		—	*Cx. ensiformis*	2
		—	*Cx. trigeminatus*	1
		—	*Cx. zeteki*	2
	Bastagarius	Bastagarius	*Cx. bastagarius*	3
		Iolambdis	*Cx. corentynensis*	1
	Conspirator	—	*Cx. aliciae*	4
		—	*Cx. dyius*	3
		—	*Cx.* sp. nr. *aliciae*	2
	Distinguendus	Putumayensis	*Cx. putumayensis*	2
	Educator	—	*Cx. eknomios*	3
		—	*Cx. theobaldi*	3
		—	*Cx. vaxus*	5
		—	*Cx.* sp. nr. *theobaldi*	1
	Erraticus	Erraticus	*Cx. aureonotatus*	2
			*Cx.* sp. nr. *aureonotatus*	3
		Clarki	*Cx. clarki*	2
	Evansae	—	*Cx. evansae*	1
	Inhibitator	Inhibitator	*Cx. oedipus*	2
			*Cx. rabelloi*	1
			*Cx.* sp. nr. *rabelloi*	3
			*Cx.* sp. nr. *inhibitator*	1
		Egcymon	*Cx. serratimarge*	1
	Intrincatus	Eastor	*Cx. eastor*	4
		Intrincatus	*Cx. intrincatus*	8
			*Cx. misionensis*	3
		Idottus	*Cx. idottus*	1
	Pilosus	Caudelli	*Cx. alogistus*	1
			*Cx. lacertosus*	1
		Pilosus	*Cx. pilosus*	2
Spissipes				
	Crybda	Pedroi	*Cx. crybda*	6
			*Cx.* sp. nr. *pedroi*	3
		Pereyrai	*Cx. pereyrai*	2
	Faurani		*Cx. faurani*	1
	Spissipes	—	*Cx*. *spissipes*	3
		—	*Cx*. sp. nr. *spissipes*	1
	Taeniopus		*Cx. akritos*	1
	Vomerifer		*Cx. gnomatos*	2
			*Cx. sacchettae*	2
			*Cx. vomerifer*	3
			*Cx.* sp. nr. *vomerifer*	1
			*Cx*. sp. nr. *portesi*	1
			*Cx.* sp. nr. *gnomatos*	1

The specimens used in this study were either collected from larval habitats as larvae and/or pupae or obtained as adult males/females in the field. Freshly emerged or field-collected adult mosquitoes were anaesthetized with ethyl acetate and either kept individually frozen at −80°C or dried in separate minute plastic vials in silica gel. For most specimens, species identification was based on the anatomical characters of the male genitalia, using the illustrations available in Pecor *et al*. [1], and fourth-instar larvae and pupae [15], when available. For some specimens, identification was based on the external morphology of field-collected females using Sirivanakarn's identification keys [15]. All males had their corresponding genitalia dissected and mounted on microscope slides, which are deposited in the Coleção Entomológica de Referência, Universidade de São Paulo, Brazil (FSP-USP) as vouchers.

### 2.2. Molecular methods and phylogenetic reconstruction

#### 2.2.1. Molecular markers

In the study, DNA sequence data were generated for two single-copy protein-coding nuclear genes (carbamoyl-phosphate synthetase 2, aspartate transcarbamylase, and dihydroorotase (*CAD*)) and hunchback (*HB*) and the barcode region of the mitochondrial cytochrome oxidase I (*COI*) protein-coding gene. The *CAD* locus encodes enzymes that catalyse pyrimidine biosynthesis [26], the carbamoyl-phosphate synthetase, aspartate transcarbamylase and dihydroorotase [27]. Previous studies addressing different groups of insects have used *CAD* [26–28]. *HB* is a gap gene [41] that is involved in anterior–posterior polarity determination in embryos [42,43]. It encodes a C_2_H_2_ zinc finger transcription factor whose primary function is to regulate the expression of other genes involved in embryo development. *COI* is a mitochondrial gene that is involved in electron transport. It contains a combination of highly conserved and variable regions that makes it useful for different genetic studies for metazoan species, especially as an identification tool [35,36,44,45].

#### 2.2.2. Experimental procedures

Genomic DNA extractions were performed following a salting out protocol [46] (electronic supplementary information, protocol salting out). DNA was retained at −20°C. Fragments of the *COI*, *CAD* and *HB* genes were amplified using primers listed in the electronic supplementary material, table S2. Polymerase chain reactions (PCRs) were performed with a Mastercycler Ep Gradient 5341 (Eppendorf); PCR and thermo-cycler profiles are listed in the electronic supplementary material, table S3.

PCR products for *COI* and *CAD* were purified using the polyethylene glycol (PEG) precipitation method (20% polyethylene glycol 8000/2.5 M NaCl) (supplementary data, protocol for precipitation of protein and cell debris). A commercial kit by Zymo Research (DNA Clean & Concentrator™-5; CA, USA) was employed to purify the PCR products generated for the *HB* gene. PCR products of all genes were electrophoresed in 1% Tris–acetate (TAE)–ethylenediamine tetraacetic acid (EDTA) (TAE EDTA) agarose gels stained with GelRedTM^®^ Nucleic Acid Gel Stain (Biotium Inc., Hayward, USA).

Sequencing reactions were carried out in both directions using the ABI BigDye™ Terminator v.3.1 cycle sequencing kit (PE Applied Biosystems, Warrington, UK). For sequencing the *COI*, we employed the same set of primers used for PCR, whereas for the *CAD* gene we used the M13 primers that were added to the PCR primers only. For *HB*, we performed several strategies that involved three different sets of primers; thus, when the M13 tailed primers were used for PCR reactions, the sequencing for *HB* used only the M13 (electronic supplementary material, table S2). Sequencing reactions were carried out in a total volume of 10 µl, containing approximately 20 ng of the PEG-purified PCR product; 0.5 µl BigDye Terminator ready reaction mix; 1× sequencing buffer (Applied Biosystems); 3.6 pmol of each primer (for different genes); and the remaining volume of sterile purified water. All PCR products were purified with Sephadex^®^ G-50 columns (GE Healthcare, Sigma-Aldrich, St. Louis, MO, USA). Capillary electrophoresis was performed in an Applied Biosystems 3130 DNA Analyser (Applied Biosystems, Foster City, CA, USA). Newly generated sequences for *CAD* and *HB* are deposited in GenBank under accession codes MG703269–MG703382 (*CAD*) and MG703383–MG703481 (*HB*). The *COI* barcode sequences are available in GenBank under accession codes KX779776–KX826818 and were first published in [40].

#### 2.2.3. Sequence analyses

Sequences were manually edited using Geneious version R 9.0 (Biomatters Ltd, Auckland, New Zealand) and Mesquite v3.2 [47]. Primer sequence regions were removed from all sequences. The chromatograms of each DNA sequence were visualized to verify which sequences were optimal and which needed to be re-processed, and BLASTed (using *megablast*, *blastn* and *blastx*) to sequences in NCBI's NR database and to the *Cx. quinquefasciatus* reference genome to confirm homology [48]. Polymorphic sites in the single-copy nuclear genes were coded as ambiguous sites. Sequence alignments were done for both nucleotides and amino acids for each of the three datasets. All alignments were made using Clustal W, implemented in Geneious version R 9.0 (default parameters), and adjusted manually to remove alignment artefacts.

The dataset was further explored following four steps: (i) the best-fitting model of nucleotide substitution and frequencies was determined using jModeltest version 2 (considering the Akaike information criterion) [49]; (ii) distance matrices, number of variable sites, conserved sites and parsimony informative sites were determined using MEGA v. 6.0.6 [50]; (iii) likelihood mapping analysis was implemented with TREE PUZZLE to address the information content of each dataset as described by Strimmer & von Haeseler [51]; and (iv) DNA sequences were translated to amino acid sequences to verify their identity (expected protein according to the target gene) and to verify coding sequences and absence of stop codons.

#### 2.2.4. Phylogenetic analysis

DNA sequences were aligned and then concatenated to make a super-matrix. Only individuals with a complete set of three genes were employed in the analyses. The *COI* dataset was also analysed separately with all individuals that had a *COI* sequence available, and the results can be found in [40]. Given that *CAD* was obtained for a greater number of specimens than the *HB* gene, the phylogenetic trees based solely on *CAD* sequences from all individuals are shown in figure 4 (BA topology) and figure S1 (ML topology in electronic supplementary data).

Maximum-likelihood (ML) inference was performed with PhyML (v3.0 [51]) with the approximate likelihood ratio (aLRT-SH) as support values [52], employing the concatenated data matrix of *COI*, *CAD* and *HB* genes. The nucleotide substitution model used for the nuclear genes was selected using jModeltest. Considering the limited number of models available in PhyML, the TIM2+I+G best-fitting model chosen for *CAD* by jModeltest was replaced by the GTR+I+G in the PhyML analysis (electronic supplementary material, figure S1). Optimization of the search for the ML tree used the Best of the nearest neighbour interchange and the Subtree Pruning Regraftin SPR in PhyML.

Bayesian analysis (BA) was performed with MrBayes v.3.2 [53] using the concatenated dataset of *CAD* + *HB* + *COI* and *CAD* genes. Datasets were partitioned by gene following distinct nucleotide substitution models chosen using jModeltest; all codons were included in the analysis. Parameters chosen for the estimation of the posterior probability were (i) GTR as the substitution model for nucleotides with a γ-distributed rate variation across sites and (ii) a proportion of invariable sites. Parameters were unlinked between all partitions and different transition matrices; proportion of invariant sites and different state frequencies were used. The BA was run for 2 000 000 generations, using four chains. Chains were sampled every 2500 generations, discarding the first 25% of the topologies sampled. The consensus tree was generated using the ‘allcompat’ option.

## 3. Results

The length of the DNA sequence alignment was 658 base pairs (bp) for *COI* and 610 bp for the *CAD* gene. The *HB* gene sequence alignment consisted of the forward strands only because all different approaches adopted to obtain the reverse strand failed. The *HB* sequences ranged from 470 to 540 bp in length; unreliable regions were masked, leaving 483 bp in the final alignment.

**Figure 1.**
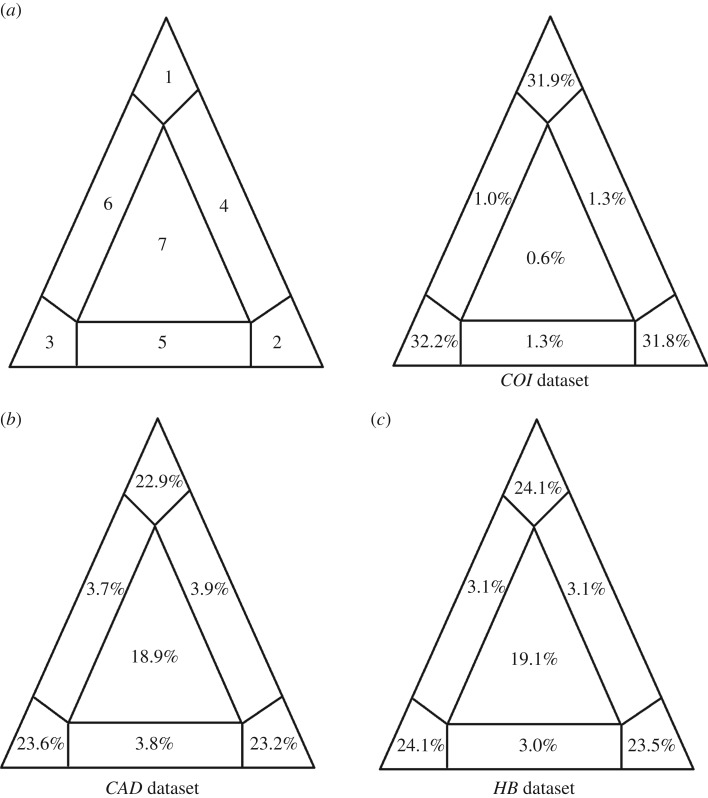
Likelihood mapping quarter distribution (Seven Basins of Attraction) for the three datasets (*COI*, *CAD*, *HB*). Estimated quarter distribution for (*a*). *COI* dataset (under HKY model); (*b*) *CAD* dataset (under GTR model); (*c*) *HB* dataset (under GTR model). The analysis corresponding to *COI* gene has been previously published in [40]. Authors have the right to reproduce this image.

The results of a likelihood mapping analysis are shown in figure 1. The triangles include different quarters showing the partly resolved, unresolved and fully resolved quarters for each separate dataset. Fully resolved quarters, indicated by regions 1, 2 and 3, correspond to moderately high percentages for all three single-gene alignments (*COI*: 95.9%, *CAD*: 69.7% and *HB*: 71.7%), showing that there is good signal in the data. The partly resolved quarters and unresolved quarters were low for all datasets.

**Figure 2.**
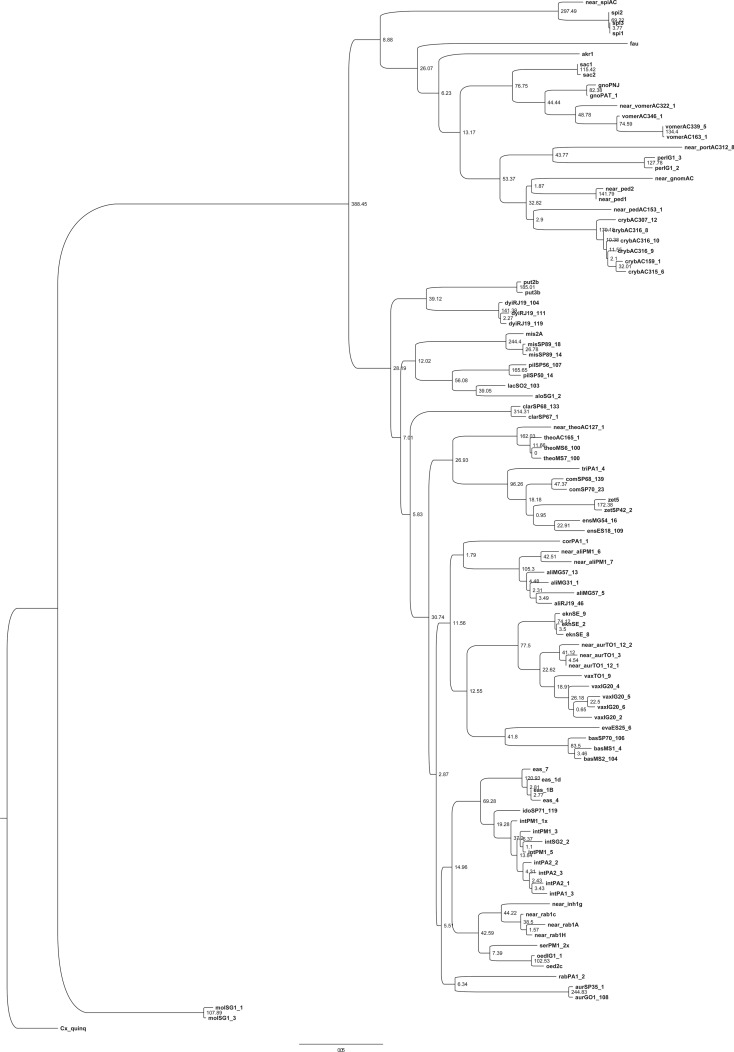
Maximum-likelihood-based phylogenetic tree obtained from a concatenated matrix (*COI *+ *CAD *+ *HB* matrix) of *Culex* (*Melanoconion*) species, with aLRT statistic values shown as node support.

The ML tree (figure 2), based on the available DNA sequence data, supported the monophyly of the Spissipes (aLRT = 8.88) and Melanoconion (aLRT = 28.19) sections within the subgenus. Specimens identified as *Cx*. *spissipes* and *Cx*. sp. nr. *spissipes* clustered together in a clade sister to remaining species of the Spissipes Section. Furthermore, unexpected groupings of species were found within the two sections, demonstrating that the current subdivision into groups and subgroups, based primarily on male genitalia traits, does not correspond to the relationships recovered from the ML analysis of this molecular data. The monophyly of the Vomerifer and Crybda Groups was not confirmed because *Cx.* sp. nr. *portesi* was found to be a sister to *Cx. pereyrai* of the Crybda Group, and the single specimens of *Cx*. sp. nr. *gnomatos* included in the analyses was recovered in a clade that includes *Cx.* sp. nr. *pedroi* and *Cx. crybda* of the Crybda Group. In the ML tree, most of the species of the Melanoconion Section displayed a different arrangement from the current classification, with the exception of the Pilosus Group (*Cx. pilosus*, *Cx. alogistus* and *Cx. lacertosus*) and Atratus Group (*Cx. trigeminatus, Cx. ensiformis, Cx. zeteki*, *Cx. commevynensis*; *Cx. dunni*), which were recovered as monophyletic groups.

**Figure 3.**
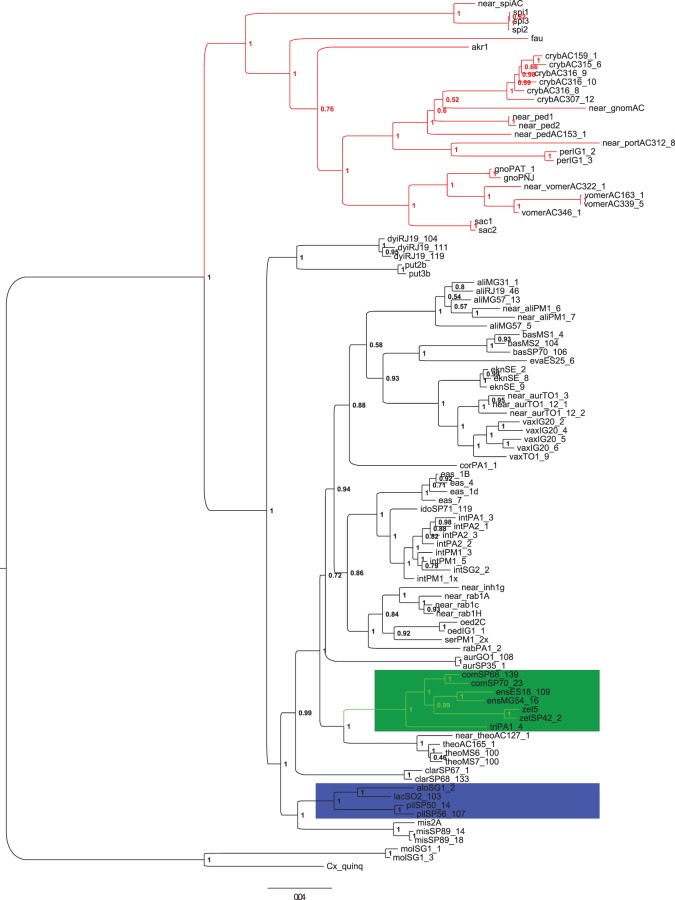
Inferred topology from the Bayesian analysis of a concatenated matrix (*COI *+ *CAD *+ *HB*) of *Culex* (*Melanoconion*) species, with posterior probability values shown as node support. The Spissipes Section (red) and the monophyletic groups of the Melanoconion Section, Pilosus Group (blue) and Atratus Group (green) are highlighted.

**Figure 4.**
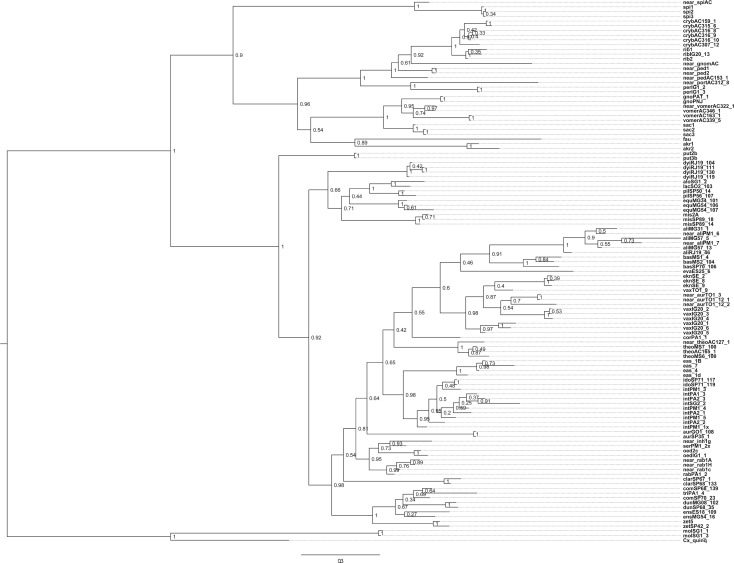
Inferred topology from the Bayesian analysis of the *CAD* dataset of *Culex* (*Melanoconion*) species, with posterior probability values shown as node support.

The results of BAs (figure 3) were highly consistent with those found in the ML analysis, showing two major sister groups that correspond to the Spissipes and Melanoconion Sections. The Bayesian posterior probabilities for the groups were strong (BPP = 1). Each major branch included multiple groups and subgroups, which were previously recognized in the classification of the subgenus. However, the Bayesian tree did not support the Vomerifer and the Crybda Groups because the single specimens of *Cx*. sp. nr. *gnomatos* and *Cx*. sp. nr. *portesi* were into the clade composed of species of the Crybda Group. The BA topology recovered clades (BPP = 1) from which only two were consistent with groups that were defined based on morphological similarities, specifically the Atratus and Pilosus Groups, which were recovered in both the BA (figure 3) and the ML (figure 2) trees. Results of the Bayesian (figure 4) and ML analyses (electronic supplementary material, figure S1) based only on the DNA sequences of the *CAD* nuclear gene were consistent in showing the Crybda Group of the Spissipes Section as a polyphyletic lineage ((*Cx. crybda* + *Cx. ribeirensi*s) + *Cx*. sp. nr. *gnomatos* + *Cx.* sp. nr. *pedroi* + (*Cx.* sp. nr. *portesi* + *Cx. pereyrai*)).

Other monophyletic clades were recovered within the Melanoconion Section in the ML tree (figure 2), and their placements were consistent with the groupings recovered in the Bayesian tree (figure 3). For instance: (1) *Cx. aliciae* is placed as a sister to (*Cx. bastagarius* + *Cx. evansae*) + (*Cx. eknomios* + *Cx. vaxus*) + *Cx. corentynensis*. (2) A cluster comprised (*Cx. eastor*, *Cx. idottus* and *Cx. intrincatus*), as a sister to (*Cx.* sp. nr. *inhibitator*, *Cx.* sp. nr. *rabelloi*, *Cx. oedipus*, *Cx. serratimarge* and *Cx. rabelloi*) (BPP = 0.86). *Culex rabelloi* of the Inhibitator Group was recovered outside the clade composed of (*Cx.* sp. nr. *inhibitator*, *Cx.* sp. nr. *rabelloi*, *Cx. oedipus*, *Cx. serratimarge*) in the ML tree (figure 2), whereas in the Bayesian tree (figure 3) *Cx*. *rabelloi* was recovered together with the remaining species of the Inhibitator Group. (3) In both Bayesian and ML topologies, *Cx. aurenotatus* and *Cx*. sp. nr. *aureonotatus* within the Melanoconion Section do not form a group. Specimens identified as *Cx*. sp. nr. *aureonotatus* were recovered within the clade composed of *Cx*. *eknomios* and *Cx*. *vaxus*, whereas *Cx*. *aureonotatus* GO1_108 and *Cx*. *aureonotatus* SP35_1 were in a separate clade, depending on the analytical approach adopted. In the Bayesian topology (figure 3), *Cx. aurenotatus* was recovered as a sister of a major clade that includes species of the Conspirator, Bastagarius, Evansae, Educator, Intrincatus and Inhibitator groups. In the ML topology (figure 2), *Cx. aureonotatus* GO1_108 and *Cx*. *aureonotatus* SP35_1 and *Cx. rabelloi* PA1_2 were recovered as sisters (aLRT = 6.34), 4) within a clade that includes species of the Intrincatus and Inhibitator groups. A few clades were consistent in both the Bayesian and ML topologies, with *Cx. misionensis* sister to the Pilosus Group and the placement of *Cx. theobaldi* and *Cx*. sp. nr. *theobaldi* as sister to the Atratus Group, with a high Bayesian posterior probability.

The relationships of the sampled species in most of the informal groups currently recognized in the Melanoconion Section had a clustering pattern different from the known classification based on morphology. In all the recovered topologies, *Cx. aliciae* and *Cx. dyius* (representatives of the Conspirator Group) were recovered as branches of different clades. Similarly, *Cx. clarki* and *Cx. aurenotatus* (representatives of the Erraticus Group) were recovered as members of different clades.

## 4. Discussion

DNA sequence data available for the subgenus *Melanoconion* are limited; however, a few recent studies using ribosomal and mitochondrial DNA can be highlighted. For instance, genetic variation among *Cx. erraticus* from Colombia, Guatemala and nine locations in the USA have been addressed using the ND4 mitochondrial gene and the ITS2 region of rDNA [54]. The results of analyses revealed two major lineages within the species. One lineage included central and eastern US populations, whereas the other corresponded to Central America, South America and the western USA, with little gene flow among populations from distinct geographical regions. Focusing on species identification, William & Savage [55] were able to identify eight species of the subgenus *Melanoconion* that occur in the southern USA using multiplex PCR of the small subunit of the ribosomal RNA *18S* gene. Phylogenetic relationships within the Vomerifer and Pedroi Groups of the Spissipes Section have been demonstrated to be monophyletic sister groups using the internal transcribed spacer (ITS2) of ribosomal RNA. *Culex* *adamesi*, *Cx.* *ribeirensis* and *Cx.* *pedroi* clustered together, indicating the monophyly of the Crybda Group. In addition, the monophyly of the Vomerifer Group has been corroborated, and the presence of two divergent branches suggests that the name *Cx.* *pedroi* can include two species. Vesgueiro *et al*. [19] using ITS-2 sequence data of several species of the subgenera *Culex*, *Melanoconion* and *Phenacomyia* showed the difficulties one may face in obtaining and aligning the ITS-2 sequences for studies aimed at assessing relationships among *Culex* species because of high intraspecific variation. In addition, the results of analyses suggested that the name *Cx. aliciae* includes at least two morphologically similar species, and that the Conspirator Group that includes *Cx. dyius* and *Cx. aliciae* among other species is not monophyletic, with *Cx. ybarmis* of the Intrincatus Subgroup of the Intrincatus Group within the branch composed of *Cx. aliciae*.

The Spissipes and Melanoconion Sections can be recognized by the morphological features of the male genitalia using the available illustrations in [1], female fourth-instar larvae and pupae based on the identification keys of Sirivanakarn [15]. Morphological traits used in Sirivanakarn's identification keys may represent synapomorphies for each section; however, they need to be tested in further analyses of the whole group, including other *Culex* subgenera. According to Sallum & Forattini [2] and Sirivanakarn [15], species of the Spissipes Section can be distinguished from those of the Melanoconion Section based on the shape and distribution of decumbent, narrow and broad scales on the vertex and occiput of both males and females, and the shape of the aedeagus and its connections to the lateral plates of the male genitalia (narrow and curved in the Melanoconion Section and broad and curved in the Spissipes Section, in lateral view). Based on fourth-instar larvae, both sections can be distinguished by the shape of the siphon, siphonal index and distribution of the siphonal setae, whereas seta 8-VIII arises from the angle of segment VIII in the Spissipes Section, but it is displaced anteroventrally in species of the Melanoconion Section. The results of the current analyses provide support for the monophyly of both the Spissipes and Melanoconion Sections. Moreover, our results are consistent with most of the morphological classification of the Spissipes Section; while the relationships within the Melanoconion Section are somewhat incongruent with morphology-based groups. The incongruence might be caused by the small percentage of taxa of the Melanoconion Section included in the study.

Currently the Spissipes Section includes 23 species separated into eight groups and three subgroups [2]. Results of the analyses of the concatenated three-gene data matrix did not support the monophyly of the Vomerifer and Crybda Groups. However, it is plausible to hypothesize that the specimens identified as *Cx*. sp. nr. *gnomatos* and *Cx*. sp. nr. *portesi* using morphological similarity of the females might belong to as-yet undescribed species that belong to those two groups. There are undescribed *Melanoconion* species that have been mentioned by Linton *et al*. [56] and Hutchings *et al*. [57] in areas of the Amazon river basin. Some of them are morphologically similar to species of the Spissipes Section. Considering that the classification into groups and subgroups depends primarily on features of the male genitalia, the identification of *Cx*. sp. nr. *portesi* and *Cx*. sp. nr. *gnomatos* was preliminary as it was based on the external morphology of field-collected females. The informal internal classification of the Melanoconion Section is challenging. This section includes 137 species, and the last revision based on morphology is that by Sirivanakarn in 1983 [15]. Even though the relationships within the Melanoconion Section need further investigation, our results clearly support the monophyly of the section. For the Melanoconion Section, we included representatives of 10 out of 13 groups: Atratus Group, Bastagarius Group, Distinguendus Group, Conspirator Group, Educator Group, Erraticus Group, Evansae Group, Inhibitator Group, Intrincatus Group and Pilosus Group (table 1). The Peccator, Saramaccensis and Trifidus Groups were not represented in the study. The results of the analyses found support for monophyly of the Atratus and Pilosus groups. All other groups included in the study were found to be polyphyletic or paraphyletic. The ML and Bayesian statistical support for the monophyly of the Pilosus and Atratus groups is strong and consistent independent of the method of analysis that was used and the dataset employed for the analyses. The results of this study open a discussion regarding the internal classification of the Melanoconion Section, because they do not give conclusive answers as the taxon representation of section was limited.

Moreover, the results of the analyses demonstrate that the current hypothesis of classification of the Spissipes and Melanoconion Sections may require rearrangement to reflect the monophyletic groups within each section. The ML and Bayesian topologies based on the three concatenated genes recovered morphology-based groups and thus any future change should consider these findings. According to the current classification, the Intrincatus Group consists of 25 species arranged into six subgroups. In the current study, four species were included (five species in the *CAD* only topology), which formed two separate groups. The support for (*Cx. eastor, Cx. idottus* and *Cx. intrincatus*) is high (aLRT = 69.28 and BPP = 1), indicating a smaller group of closely related species. Other species of the Intrincatus Group (*Cx. misionensis* and *Cx. equinoxialis*—the latter in the *CAD* only topology) did not nest together to support the monophyly of the group. It is important to conduct further studies with better taxon sampling and other high-resolution molecular sequence data to resolve the relationships of species of the Spissipes and Melanoconion Sections.

The monophyly of several morphology-based groups was not supported by our results, such as the Vomerifer, Crybda, Conspirator, Erraticus, Educator, Inhibitator and Bastagarius groups. All these groups need further investigation with a greater representation. As has been documented for other insect groups [34,58], combining morphological and molecular data may solve problematic relationships similar to those we found for the Melanoconion Section. Despite the existence of an illustrated catalogue of the species of the subgenus *Melanoconion* [1], and keys to the informal categories included in this Section [15], reaching an accurate identification of the species remains a challenge for entomologists who are not familiar with the variety of form and structures that are used to identify species of the subgenus. In 1950, Rozeboom & Komp [59] published the first revision on the subgenus and provided an identification key that greatly improved the knowledge about the subgenus and helped entomologists to recognize new species. More recently, Pecor *et al*. [1] published a catalogue with hundreds of illustrations of male genitalia and taxonomic information for each *Melanoconion* species known at the time. It is noteworthy that, in the catalogue, the authors provided illustrations of the senior and junior synonyms for each species; however, these were from distinct geographical localities. Unfortunately, the lack of a taxonomical revision of the Melanoconion Section that includes detailed descriptions and identification keys for all life stages leaves a huge gap that should be overcome in future investigations.

The ML and Bayesian analyses of the multi-loci dataset were highly informative and consistent in showing the monophyly for both sections of the subgenus and recovering most of the groupings currently known for the Spissipes Section. Moreover, our findings coupled with additional studies should lead to an improved classification of the Melanoconion Section, as we recovered some morphology-based groups and determined which groups need further study. We presented here the most inclusive molecular assessment of relationships within the subgenus *Melanoconion,* with evidence to support both sections as natural groups

## 5. Conclusion

Our study found support for the two major sections of the subgenus *Melanoconion*, the Spissipes and the Melanoconion sections. The Vomerifer and the Crybda groups of the Spissipes Section may not be monophyletic groups. Consequently, further investigation is required because the specimens of *Cx*. sp. nr. *gnomatos* and *Cx*. sp. nr. *portesi* were field-collected females that may have been misidentified due to constraints in identifying adult females in this group. It will be of fundamental importance to have male and female specimens linked to larvae and pupae for an accurate identification and to expand the currently available keys for this group. Based on the current classification of the subgenus, a few groups and subgroups were recovered as natural groups. This finding highlights the need for further investigations using other genes, in addition to morphological data and more representative taxon sampling of all groups and subgroups currently included in the subgenus. Our data showed that the *CAD* gene may be used as an additional marker for assessing relationships within *Culex* (*Melanoconion*), and possibly other *Culex* subgenera.

## Supplementary Material

Supplementary data S1

## Supplementary Material

Supplementary data S2

## Supplementary Material

Supplementary Table S3

## Supplementary Material

Supplementary information

## Supplementary Material

Figure S1

## Supplementary Material

Figure S2

## Data accessibility

This manuscript comprises three supplementary data files containing the complete information of all specimens included in the study: geographical origin, corresponding codes in the entomological collection of the Faculdade de Saúde Pública de São Paulo, Brazil and all GenBank accessions (*COI*, KX779776–KX779895 and KX826817–KX826818; *CAD*, MG703269–MG703382; *HB*, MG703383–MG703481 and MG787083–MG787084). Sequences for *Culex quinquefasciatus* were downloaded from GenBank, *COI*, HQ398883, *CAD*, XM_001848657; and the sequence for *HB* was obtained in this study and is available in GenBank with accession number MH023218. Additional figures obtained during the phylogenetic analyses (electronic supplementary material, figure S1; Figure S2) have been included as supplementary data to present the phyML topologies for *CAD* and *CAD*+*HB*, as additional support for our hypotheses.
