# Supplementary material for: Molecular phylogeny of Culex subgenus Melanoconion (Diptera: Culicidae) based on nuclear and mitochondrial protein-coding genes
Source: R Soc Open Sci. 2018 May 23;5(5):171900. doi: 10.1098/rsos.171900 (PMC5990733; doi:10.1098/rsos.171900)
Supplement: Supplementary data S2 [file rsos171900supp2.docx]

Table S2. List of primers used for PCR amplification and sequencing of the mitochondrial and nuclear protein coding genes of *Culex* species.

| **Gene** | **Primer used for F strand** | **Primer used for R strand** |
| --- | --- | --- |
| *COI* ^a^ | LCO1490_F  5'-ggt caa caa atc ata aag ata ttg g-3' | HCO 2198_R  5'-taa act tca ggg tga cca aaa aat ca-3' |
| *CAD*^b^ | *CAD*m-F  5´- tgt aaa acg acg gcc agt gtn gtn aar atg ccn mgn tgg ga-3´ | *CAD*m-R  5´- cag gaa aca gct atg acc cai cci aci gcr cac car tcr aay tc  -3´ |
| *HB*^c^ | *HB*m-F  5´- tgt aaa acg acg gcc agt tgy cci aar tgy cci tty gti aci g -3´ | *HB*mR-  5´- cag gaa aca gct atg acc gcy tgy tgr tci gcr aac aty tgr a –3´ |
| *HB*^d^ | - | *HB*mR2-  5´- gtt gyt kgw ggc bgc ryt ytt gaa c -3´ |

^a^ Folmer et al. (1994) [44].

^b^ Primers used by Reidenbach et al. (2009) [34], herein modified with the addition of M13 sequence (marked in grey color).

^c^ Primers used by Reidenbach et al. (2009) [34], herein modified with the addition of M13 sequence (marked in grey color) [32].

^d^ Primer designed for this work targeting a region of the R strand of *HB*.
