# Supplementary material for: Molecular phylogeny of Culex subgenus Melanoconion (Diptera: Culicidae) based on nuclear and mitochondrial protein-coding genes
Source: R Soc Open Sci. 2018 May 23;5(5):171900. doi: 10.1098/rsos.171900 (PMC5990733; doi:10.1098/rsos.171900)
Supplement: Supplementary Table S3 [file rsos171900supp3.docx]

Table S3. PCR condition and thermo-cycler profile for amplification of each gene used in this work.

| **PCR reaction** | **Genes** | | |
| --- | --- | --- | --- |
|  | **COI^a^** | **CAD^b^** | **HB^c^** |
| Buffer concentration | 20 mM | 20 mM | 20 mM |
| MgCl2 concentration | 1.5mM | 2.4mM | 2.4 mM |
| F and R primer concentration | 25 pmol | 100 pmol | 112.5pmol |
| dNTPs concentration | 200 μM | 200 μM | 200 μM |
| DNA-Polymerase concentration | 0.625U | 1.25U | 1.25U |
| Purified water volume | As needed | As needed | As needed |
| E fortracted DNA volume  (1 mosquito) | 1 μL (1:20) | 2 μL | 2 - 4 μL |
| Dimethyl sulfo foride (DMSO) | - | 5% | 10% |
| Final volume of PCR reaction | 25 μL | 25 μL | 25 μL |
| PCR amplification profile | 94°C for 3min;-  35 cycles of (94°C for 1 min; 55°C for 1 min; 72°C for 1 min);  Annealing and extension - 72°C for 7 min –  Final extension. | 94°C for 2min;  40 cycles of (94°C for 30 seg; 55°C for 30 seg; 72°C for 2mins);  Annealing and extension - 72°C for 5 min -  Final extension. | 94°C for 2min;  40 cycles of (94°C for 30 seg; 55°C for 30 seg; 72°C for 2min);  Annealing and extension - 72°C for 5 min -  Final extension. |

^a^ For *COI* reactions we used Platinum® *Taq* DNA Polymerase, High Fidelity (Life Technologies).

^b^ For *CAD* reactions we used GoTaq® DNA Polymerase (Promega).

^c^ For *HB* reactions we used GoTaq® DNA Polymerase (Promega).
