# Supplementary material for: Molecular phylogeny of Culex subgenus Melanoconion (Diptera: Culicidae) based on nuclear and mitochondrial protein-coding genes
Source: R Soc Open Sci. 2018 May 23;5(5):171900. doi: 10.1098/rsos.171900 (PMC5990733; doi:10.1098/rsos.171900)
Supplement: Supplementary information [file rsos171900supp4.docx]

**Electronic Supplemental information for “Molecular phylogeny of *Culex* subgenus *Melanoconion* (Diptera: Culicidae) based on nuclear and mitochondrial protein coding genes”**

**Laboratory protocols**

**Protocol 1 - DNA extraction - Salting Out**

Source: Modified from Miller et al. (1988).

**Part 1**

- Tissue Digestion

1. Macerate each mosquito inside a 1.5 mL tube with 300 µL of TNES buffer (TNES buffer: 10 mM Tris-HCL, pH 7.5, 400 Mm NaCl, 100 mM EDTA pH 8.0, 0.6% SDS).

2. Add 1μL Proteinase K (18.7 mg/ml) to each tube (with a macerated mosquito in TNES buffer).

3. Incubate at 56°C for 16-18 hours (overnight) in a dry block heater with agitation (600 rpm per 15 sec every 10 mins).

- Precipitation of Protein and Cell Debris

4. Add 85 µL NaCl (5M) to each tube and homogenize.

5. Centrifuge at 13,000 rpm for 10 mins at ambient temperature.

6. Transfer supernatant into a new tube.

- Precipitation of Nucleic Acids

7. Add 300 µL of cold 98% ethanol (4°C) to each tube and homogenize by inverting the tubes upside down, carefully.

8. Incubate at -20°C for ~20 minutes.

9. Centrifuge (preferably at 4°C) at 13,000 rpm in a benchtop centrifuge for 10 minutes.

10. Flip the tube carefully to discard the supernatant.

11. Add 500 µL of 70% ethanol and repeat from step 3 (Centrifuge).

12. Allow to air dry inside a speed vacuum for 5 to 10 mins at 60°C.

13. Resuspend in 100 µL of TE buffer.

**Part 2**

- Removing RNA

1. Add 0.1 µL RNAse (10 mg/ml) to every 10 µL of DNA product of extraction protocol.

2. Incubate at 37°C for 30 mins.

3. Store all products in freezer -20°C.

**Critical steps**

- After adding sodium chloride and centrifuging be careful not to transfer any of the white solid (cell debris and SDS) into the fresh tube (Part 1, Step 4).
- Ensure to dry the pellet of DNA completely before attempting to resuspend (Part 1, Step 12).

**Supplemental tables**

Table S1. List of species of the genus Culex included in the study, specimen identification code, location, GenBank accession code of sequences used in the analyses, including those that were newly sequenced.

Table S2. List of primers used for PCR amplification and sequencing of the mitochondrial and nuclear protein coding gene of *Culex* species with source of information.

Table S3. PCR condition and thermo-cycler profile for amplification of each gene used in this work.

**Legends for Figure S1 and Figure S2**

**Figure S1.** *Culex* (*Melanoconion*). Phylogenetic tree using DNA sequences from the *CAD* nuclear protein coding gene of 112 specimens. Analysis used PhyML, using the GTR model. Support values are given by aLRT statistics.

**Figure S2.** *Culex* (*Melanoconion*). Phylogenetic tree of concatenated matrix of *CAD*+*HB* nuclear protein coding genes of 99 specimens. Analysis used PhyML, using the GTR model. Support values are given by aLRT statistics.
