# Supplementary figures and images for: Molecular phylogeny of Culex subgenus Melanoconion (Diptera: Culicidae) based on nuclear and mitochondrial protein-coding genes
Source: R Soc Open Sci. 2018 May 23;5(5):171900. doi: 10.1098/rsos.171900 (PMC5990733; doi:10.1098/rsos.171900)

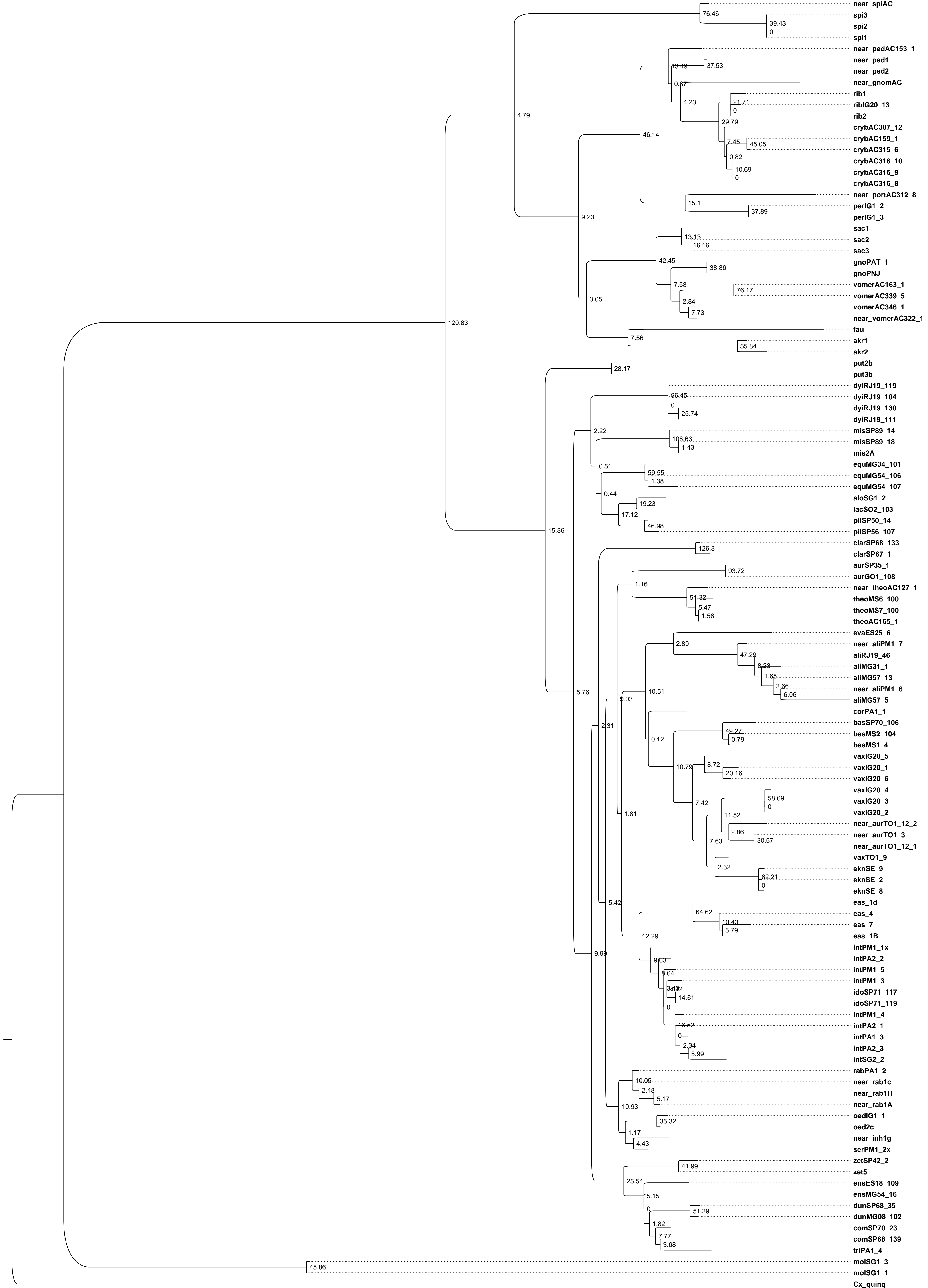

Supplement: Figure S1 [file rsos171900supp5.pdf]

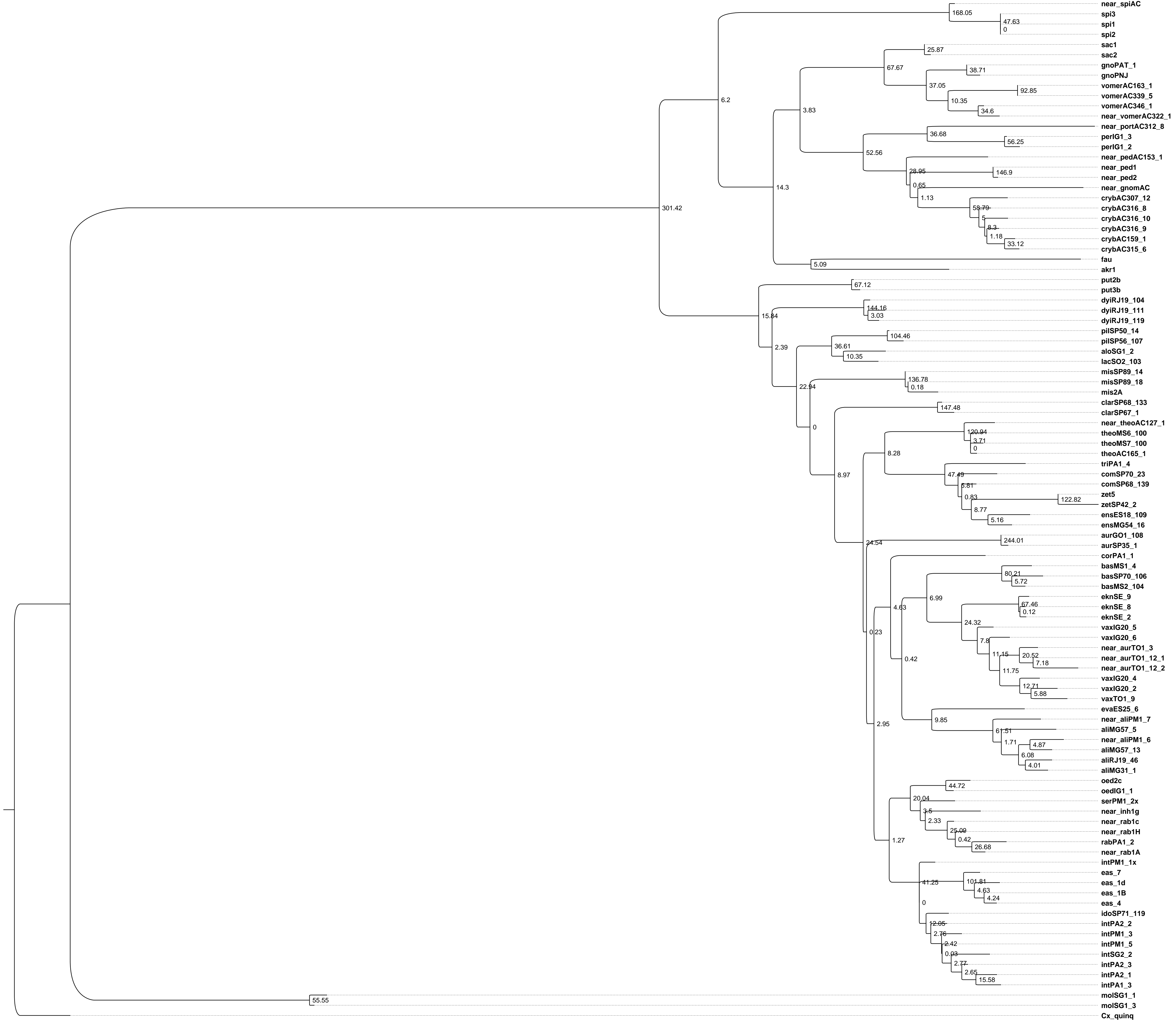

Supplement: Figure S2 [file rsos171900supp6.pdf]
